# Clinical Outcome of RAMPS for Left-Sided Pancreatic Ductal Adenocarcinoma: A Comparison of Anterior RAMPS versus Posterior RAMPS for Patients without Periadrenal Infiltration

**DOI:** 10.3390/biomedicines9101291

**Published:** 2021-09-22

**Authors:** Jaewoo Kwon, Yejong Park, Eunsung Jun, Woohyung Lee, Ki Byung Song, Jae Hoon Lee, Dae Wook Hwang, Song Cheol Kim

**Affiliations:** 1 Department of Surgery, Kangbuk Samsung Hospital, Sungkyunkwan University School of Medicine, Seoul 03181, Korea; skunlvup@naver.com; 2 Division of Hepato-Biliary and Pancreatic Surgery, Department of Surgery, University of Ulsan College of Medicine and Asan Medical Center, Seoul 05505, Korea; blackpig856@gmail.com (Y.P.); go1040@hanmail.net (E.J.); ywhnet@gmail.com (W.L.); mtsong21c@naver.com (K.B.S.); hbpsurgeon@gmail.com (J.H.L.); dwhwang@amc.seoul.kr (D.W.H.); 3 Convergence Medicine, University of Ulsan College of Medicine, Seoul 05505, Korea

**Keywords:** pancreatic cancer, RAMPS, distal pancreatectomy, adrenalectomy

## Abstract

Radical antegrade modular pancreatosplenectomy (RAMPS) is considered an effective procedure for left-sided pancreatic ductal adenocarcinoma (PDAC). However, whether there are differences in perioperative outcomes, pathologies, or survival outcomes between anterior RAMPS (aRAMPS) and posterior RAMPS (pRAMPS) has not been reported previously. We retrospectively reviewed and compared the demographic, perioperative, histopathologic, and survival data of patients who underwent aRAMPS or pRAMPS for PDAC. We also compared these two groups among patients without periadrenal infiltration or adrenal invasion. A total of 112 aRAMPS patients and 224 pRAMPS patients were evaluated. Periadrenal infiltration, neoadjuvant treatment, and concurrent vessel resection were more prevalent in the pRAMPS group. After excluding patients with periadrenal infiltration, 106 aRAMPS patients were compared with 157 pRAMPS patients. There were no significant differences between the aRAMPS and pRAMPS groups in the pathologic tumor size, resection margin, proportion of tangential margin in the R1 resection, and number of harvested lymph nodes. The median overall survival and disease-free survival also did not differ significantly between the two groups. We cautiously suggest that pRAMPS will not necessarily provide more beneficial histopathologic outcomes and survival rates for left-sided PDAC cases without periadrenal infiltration. If periadrenal infiltration is not suspected, aRAMPS alone should be sufficiently effective.

## 1. Introduction

Pancreatic ductal adenocarcinoma (PDAC) is among the most aggressive cancer types. Left-sided PDAC, which is located in the pancreatic body or tail, typically has a poor prognosis because of non-specific symptoms and consequent late presentation or delayed diagnosis [1]. Surgical resection is still a curative treatment, and distal pancreatectomy with splenectomy is the standard operative approach for left-sided PDAC. Radical antegrade modular pancreatosplenectomy (RAMPS) was first described by Strasberg [2] as a technique that provides more sufficient tangential margins and more harvested lymph nodes than standard distal pancreatectomy with splenectomy (SDP). Several studies have demonstrated that RAMPS increases the negative tangential margin rate and lymph node harvest yield [3,4,5]. However, no publications have reported on the differences between anterior radical antegrade modular pancreatosplenectomy (aRAMPS) and posterior radical antegrade modular pancreatosplenectomy (pRAMPS) in terms of tangential margin status, lymph node retrieval, and survival outcomes. In addition, to date, pRAMPS is performed at the surgeon’s discretion when periadrenal infiltration is suspected according to preoperative imaging or intraoperative observation [2,5], but no evidence exists to support or dispute this practice. Thus, it is important to identify whether there are differences between aRAMPS and pRAMPS for the treatment of left-sided PDAC. This study aimed to determine whether there is a difference between aRAMPS and pRAMPS and whether aRAMPS is an appropriate treatment for left-sided PDAC without periadrenal infiltration, by comparing pathologic outcomes, overall survival (OS), and disease-free survival (DFS) between aRAMPS and pRAMPS.

## 2. Materials and Methods

### 2.1. Patients and Postoperative Monitoring

**Figure 1:**
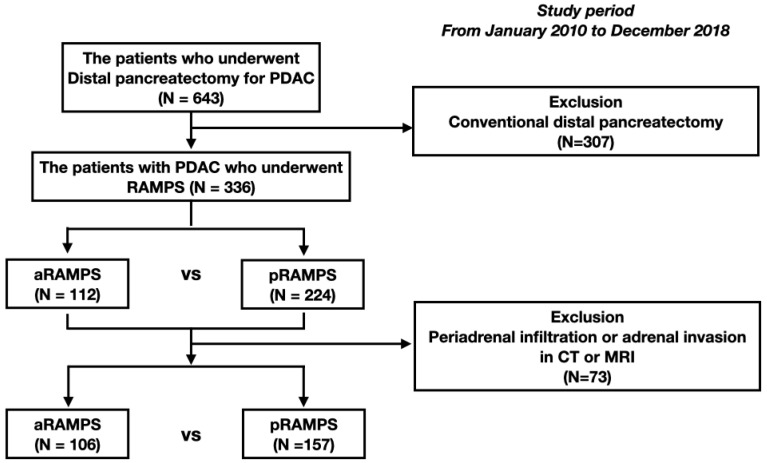
Study patient flow diagram. A retrospective review was conducted of 336 patients who underwent a distal pancreatectomy. Of these cases, 112 patients underwent aRAMPS and 224 patients underwent pRAMPS. After excluding patients with periadrenal infiltration or adrenal invasion evident on preoperative computed tomography or magnetic resonance imaging, 106 aRAMPS patients and 157 pRAMPS patients were compared.

This was a retrospective, observational cohort study. A total of 643 consecutive PDAC patients who underwent distal pancreatectomy at the Asan Medical Center between January 2010 and December 2018 were initially screened. Eight surgeons performed distal pancreatectomy for left-sided PDAC. SDP was usually performed before 2009—the year RAMPS was started in our institution—and the indications were similar to those of aRAMPS. Between 2009 and 2013, depending on the surgeon, aRAMPS and SDP were selected according to similar indications. From 2013 onward, RAMPS was selected as the main treatment for left-sided PDAC. However, depending on the surgeon’s preference, SDP might have been performed if the lesion was confined within the pancreas, peripancreatic infiltration was not suspected, and R0 resection was possible. Posterior RAMPS was performed when imaging findings suggested the presence of periadrenal infiltration (including full-fledged adrenal invasion), and aRAMPS was performed when otherwise. However, not only aRAMPS but also pRAMPS was performed even if the periadrenal infiltration was not identified when the mass was close to the adrenal gland for preventing R1 resection and expectation of better oncologic outcomes. All patients who underwent aRAMPS or pRAMPS and were managed at the study institution were eligible for inclusion. Patients treated with SDP were excluded (*n* = 307). We initially included 112 aRAMPS patients and 224 pRAMPS patients. Clinicopatholic and oncologic outcomes were compared between aRAMPS and pRAMPS cases. After that, among these 336 patients, those with adrenal gland involvement or periadrenal infiltration (*n* = 73) evident on computed tomography (CT) or magnetic resonance imaging (MRI) were next excluded to allow for subgroup comparisons of aRAMPS vs. pRAMPS among PDAC patients without periadrenal infiltration, considering that pRAMPS should be performed if periadrenal involvement is suspected. The patient selection flowchart is presented in Figure 1.

Data on the enrolled patients were obtained from the electronic medical records of our institution and were retrospectively reviewed. The clinicopathological data were collected and analyzed. The clinical tumor size was determined using preoperative, contrast-enhanced abdominoperineal CT or MRI. The collected perioperative data included the operative time, operation type, concurrent vessel and other organ resection, postoperative hospitalization, postoperative complications, the presence or absence of postoperative pancreatic fistula (POPF), 90-day mortality, adjuvant treatment, the interval between surgery and adjuvant treatment, and the first-line adjuvant chemotherapy regimen. The operation method was collected retrospectively based on the operation records, which were written by the first assistant and confirmed by the surgeon immediately after the surgery. In addition, by referring to postoperative CT data, we confirmed once again whether the operation record was accurately written. Minimally invasive operations included laparoscopic distal pancreatectomy and robotic pancreatectomy. Concurrent vessel resection was defined as intraoperative resection of a major vessel, such as the superior mesenteric vein (SMV), portal vein, celiac axis, common hepatic artery, or superior mesenteric artery (SMA). Concurrent resection of other organs referred to the stomach, colon, liver, kidneys, and other major organs except for the gallbladder and left adrenal gland. POPFs were graded according to the definition updated in 2016 by the International Study Group for Pancreatic Fistula [6]. Postoperative complications were classified according to the Clavien–Dindo classification [7]. Pathologic tumor size, TNM stage (American Joint Committee on Cancer (AJCC) Cancer Staging Manual, 8th edition), peripancreatic infiltration, resection margin and location of the involved margin, and pathologic adrenal gland invasion were collected as pathologic data. Regarding histopathological findings, the resection margin status was categorized as R0 or R1. R1 was defined when the shortest distance between the tumor and the resection margin was <1 mm. Tangential margin involvement was defined as tumor cells existing in the peripancreatic soft tissue margin, including the posterior, superior, and inferior borders of the specimen. Contrast-enhanced abdominoperineal CT was used for postoperative surveillance of all the study patients, and CA 19-9 levels were examined every 3 months for the first 2 years following surgery and then every 6 months thereafter. Recurrence was diagnosed based on the identification of new progressive lesions and elevated CA 19-9 levels. When potential recurrences were detected, ^18^F-fluorodeoxyglucose positron emission tomography (FDG-PET) or chest CT was performed, and biopsies were done to confirm recurrences if differential diagnoses were needed. The OS duration was calculated from the time of surgery until death or the national insurance loss date up until 1 September 2020. This retrospective cohort study was approved by the institutional review board of the Asan Medical Center (approval no. 2020-1596). The requirement for written informed consent was waived because of the retrospective and observational nature of the analyses.

### 2.2. Surgical Techniques

#### 2.2.1. Anterior Radical Antegrade Modular Pancreatosplenectomy

The aRAMPS technique was similar to methods previously described elsewhere [2,8]. Both minimally invasive and open surgical approaches are possible for RAMPS. Briefly, for the aRAMPS procedure, the lesser sac was penetrated by dividing the gastrocolic ligament by omentectomy. Tunneling was then performed under the pancreas, through the dissection of the inferior border of the pancreas over the SMV, until the pancreas was completely mobilized from the SMV and the portal vein. The foramen of Winslow was then opened, and the proper hepatic, common hepatic, and gastroduodenal arteries were identified. The pancreas was then encircled using umbilical tape to facilitate stapler insertion and pancreas transection. Pancreas transection was slowly performed using a linear stapler for 3 min to minimize parenchymal laceration and control bleeding. After transection of the pancreas, the splenic artery and vein were encircled and divided between locking clips. The lymph nodes on the anterior border of the common hepatic artery beside the left gastric artery and left side of the celiac axis were mobilized. If there was celiac axis or SMV invasion by a cancerous lesion, vascular resection was performed in tandem. The plane of dissection proceeded vertically, dividing the fat and soft tissue until the SMA was identified. After identifying and exposing the left side of the SMA, the dissection plane was turned to the left in an oblique plane that sloped posteriorly to the left. The anterior border of the aorta and superior border of the left renal vein were exposed and became the starting point of the inferior border of the dissection plane. During inferior plane dissection from the medial to the lateral side, the adrenal vein and left adrenal gland were identified and preserved. The dissection continued laterally, removing Gerota’s fascia. The lienorenal ligament and splenophrenic ligament were then ligated. For combined splenectomy, dissection using an energy device and clips was continued up to the gastrosplenic ligament and included the short gastric vessels.

#### 2.2.2. Posterior Radical Antegrade Modular Pancreatosplenectomy

The pRAMPS technique was similar to the aRAMPS technique and involved finding the SMA and exposing the anterior border of the aorta and superior border of the left renal vein. Dissection was continued more deeply to reach the diaphragm and retroperitoneal muscle layer. The left renal artery was identified, and the left renal vein was fully exposed during the dissection. From the medial to the lateral side, the soft tissue behind the adrenal gland and Gerota’s fascia were removed. The resected specimen included the pancreas, spleen, omentum, adrenal gland, and most of the retroperitoneal soft tissue.

### 2.3. Comparative Analysis

Demographic, perioperative, histopathologic, and oncologic data and outcomes were compared between the aRAMPS and pRAMPS groups. Continuous variables were reported as means ± standard deviations, as appropriate, and these were compared using Student’s *t*-test. Categorical variables were compared using the chi-square test, Fisher’s exact test, or the linear-by-linear association test. All tests were two sided, and a *p*-value of ≤0.05 was considered statistically significant. Kaplan–Meier survival curves were constructed to estimate both the OS and DFS rates. Survival rates between aRAMPS and pRAMPS patients were assessed using the log-rank test. After excluding patients with periadrenal infiltration, 106 aRAMPS cases were also compared with 157 pRAMPS patients using the same method. The survival curve was censored at 48 months, which is the last time point where the number of patients at risk was 10% of the total number of patients. Statistical analyses were conducted using SPSS Statistics for Windows, version 21.0 (IBM Corp., Armonk, NY, USA).

## 3. Results

The mean follow-up duration was 32.6 months (median 25.2; range 1.4–124.0 months). Table 1 presents the demographic characteristics of the 336 included patients who underwent RAMPS.

Among these patients, 112 underwent aRAMPS and 224 underwent pRAMPS. Age, sex, BMI, ASA score, CA 19-9 status, CEA status, mGPS, and tumor location were not significantly different between the aRAMPS and pRAMPS groups. The mean clinical tumor size was significantly smaller in the aRAMPS group (2.8 cm vs. 3.3 cm, *p* = 0.021). Six patients (5.4%) who underwent aRAMPS and 67 pRAMPS patients (29.9%) had suspected periadrenal infiltration or adrenal gland invasion according to preoperative imaging (*p* < 0.001). Significantly fewer patients in the aRAMPS group received neoadjuvant chemotherapy (7.1% vs. 16.1%, *p* = 0.022).

Table 2 lists the perioperative outcomes of RAMPS. The mean operative times were shorter among the aRAMPS cases (204 min vs. 228 min, *p* = 0.001).

More patients in the aRAMPS group were treated with minimally invasive surgery (71.4% vs. 54.5%, *p* = 0.003). The proportion of concurrent vessel resections was significantly lower in the aRAMPS group (5.4% vs. 13.4%, *p* = 0.025), but the rates of concurrent resections of other organs were comparable between the two groups. The mean postoperative hospital stay, incidence of surgical complications, POPF rate, and 90-day mortality rate were not significantly different between the two groups. There were no cases of adrenal insufficiency after pRAMPS, and none of the patients developed grade V complications. One patient in each RAMPS group died within 90 postoperative days due to early disease progression. The proportion of adjuvant treatments, mean period until the start of adjuvant treatment, and type of first-line adjuvant chemotherapy were not significantly different between the two groups.

Table 3 presents the pathological outcomes of the RAMPS treatments.

The mean pathological tumor size was smaller in the aRAMPS group, but the difference was not statistically significant between the two groups (3.4 cm vs. 3.8 cm, *p* = 0.128). The TNM stage, differentiation, pathologic peripancreatic infiltration rate, lymphovascular invasion rate, perineural invasion rate, mean number of harvested lymph nodes, mean number of positive lymph nodes, and positive lymph node ratio were also not significantly different between the groups. R0 resections were achieved for 76.8% of the aRAMPS cases and 77.2% of the pRAMPS cases. Among the pRAMPS cases, 13 patients (5.8%) had pathologic adrenal gland invasion. The median OS and estimated 1-, 2-, and 4-year OS rates were 29.4 months and 84.8%, 58.7%, and 32.8%, respectively, in the aRAMPS group and 28.7 months and 79.9%, 56.7%, and 29.9%, respectively, in the pRAMPS group (*p* = 0.856). The median DFS (11.5 months vs. 10.5 months, *p* = 0.859) was also not different between the aRAMPS and pRAMPS groups.

### Comparative Analysis of Perioperative, Pathologic, and Survival Outcomes between aRAMPS and pRAMPS Patients without Suspected or Observed Periadrenal Infiltration

Table 4 presents the demographic data of the two surgical groups after excluding patients with preoperative CT or MRI evidence of periadrenal infiltration or adrenal invasion.

In the analysis, 106 aRAMPS patients were compared with 157 pRAMPS cases. No factors, including the clinical tumor size (2.8 cm vs. 2.9 cm, *p* = 0.778) and neoadjuvant gtreatments (7.5% vs. 14.6%, *p* = 0.080), significantly differed between the two subgroups. Perioperative outcomes were also not significantly different between the two subgroups other than the operation time (202 min vs. 226 min, *p* = 0.002) and the concurrent vessel resection rate (5.7% vs. 14.6%, *p* = 0.022, Table 5). Among patients with concurrent vessel resection, three patients had SMV resection, one patient had celiac axis resection, and two patients had both SMV and celiac axis resection in the aRAMPS group. In the pRAMPS group, seven patients had SMV resection, nine patients had celiac axis resection, and seven patients had both SMV and celiac axis resections. However, there was no difference in the proportion of vessel resection type between the two groups (*p* = 0.109).

None of the histopathologic outcomes significantly differed between the two subgroups either (Table 6). 

**Figure 2:**
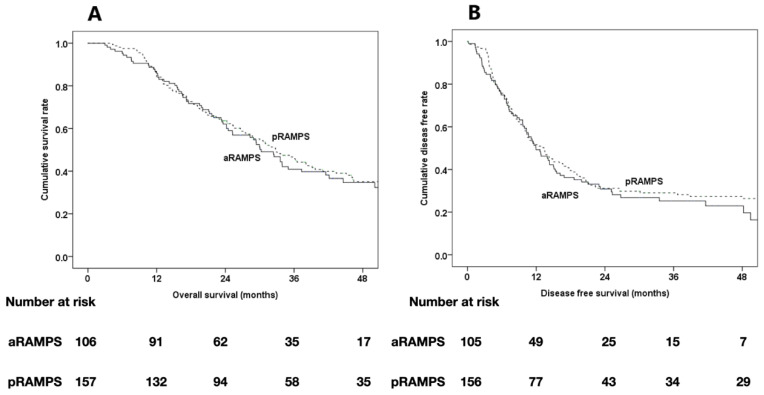
Kaplan–Meier survival curves of the cases in the aRAMPS group (*n* = 106) and the pRAMPS group (*n* = 157) without adrenal gland infiltration. (**A**) The median overall survival (OS) and estimated 1-, 2-, and 4-year OS rates were 30.3 months and 85.8%, 62.1%, and 34.7%, respectively, in the aRAMPS group and 32.8 months and 84.1%, 63.6%, and 35.1%, respectively, in the pRAMPS group (*p* = 0.318). (**B**) The median disease-free survival (DFS) and estimated 1-, 2-, and 4-year DFS rates were 12.0 months and 49.3%, 30.8%, and 22.9%, respectively, in the aRAMPS group and 13.3 months and 51.6%, 31.2%, and 27.3%, respectively, in the pRAMPS group (*p* = 0.534).

The pathologic tumor size was not different between the two groups (3.4 cm vs. 3.4 cm, *p* = 0.909). The mean numbers of harvested lymph nodes (15.1 vs. 17.0, *p* = 0.118) were not significantly different between the subgroups. R0 resection was achieved in 77.4% of aRAMPS and 80.9% of pRAMPS patients, but this difference was not statistically significant (*p* = 0.487). Among the R1 resection cases, the proportions of patients with positive tangential margins were also not significantly different between the two subgroups (91.7% vs. 96.7%, *p =* 0.519). Only one patient (0.6%) showed pathologic adrenal invasion in the pRAMPS subgroup of patients without periadrenal infiltration. The median OS (30.3 months vs. 32.8 months, *p* = 0.318), DFS (12.0 months vs. 13.3 months, *p* = 0.534), and site of recurrence (*p* = 0.463) were not statistically different between the two subgroups (Table 7, Figure 2).

## 4. Discussion

RAMPS was originally described by Strasberg et al. [2] in 2003 as a treatment for left-sided pancreatic cancer. The development of this procedure took account of the lymphatic drainage of the pancreas, reported by O’Morchoe [9], and the posterior resection margin of the pancreas and has been increasingly used as a surgical intervention for pancreatic cancer. However, there is still no definite guideline that RAMPS should be performed for left-sided PDAC. There have been several reports that RAMPS is more effective than SDP for N1 lymph node dissection and for creating a negative tangential margin in pancreatic cancer [5,10,11], but there are papers that have reported no differences in R0 resection between SDP and RAMPS [5,12]. In addition, although there is a pathological benefit in the RAMPS procedure, it is still controversial whether there is a definite advantage in the survival rate [3,5,13,14]. Most of the studies published so far have been retrospective studies, and the results were presented through a comparison between before and after the start of RAMPS [3,4,5]. As such, the indication and survival outcomes for RAMPS are not clear, and selection criteria for SDP or RAMPS may differ depending on the surgeon. In the current institution, some surgeons perform SDP if the lesion is confined within the pancreas and there is absence of peripancreatic infiltration, because R0 resection is also possible by removing the soft tissue around the pancreas while performing SDP. Although the benefits of RAMPS continue to be published and the application rate of RAMPS is increasing, more evidence is needed to determine whether RAMPS should be performed in all left-sided PDAC patients.

From this perspective, when performing RAMPS, the selection criteria of aRAMPS and pRAMPS are not yet clear. Previous studies have indicated that the decision to perform aRAMPS or pRAMPS depends on the presence or absence of adrenal gland invasion. Strasberg et al. reported that the posterior extent of invasion on a CT image determines whether the posterior plane of dissection is anterior or posterior to the adrenal gland [2]. Mitchem et al. suggested that the decision to perform aRAMPS vs. pRAMPS should be based on the position of the tumor, as assessed by preoperative CT [10]. However, it may not be accurate to determine the dissection plane by simply judging whether a cancerous invasion is evident on a CT scan. Moreover, no randomized clinical trials have been performed to provide definitive evidence for performing pRAMPS. Accordingly, there were several patients managed at our institution who underwent pRAMPS and R0 resection for better oncologic outcomes even if their periadrenal infiltration status was not clear. This preference can be seen by the higher proportion of neoadjuvant treatment before surgery in the pRAMPS group, even if it is not statistically significant. As reported by several studies [4,10], when both aRAMPS and pRAMPS are performed, the anterior renal fascia is removed. Since the adrenal gland is attached to the underside of the anterior renal fascia, it is unclear whether removal of the adrenal gland should occur if periadrenal infiltration is detected solely by preoperative imaging. Additionally, to achieve a negative tangential margin and optimize lymph node retrieval, complications—such as adrenal insufficiency and associated diseases—should be considered for any PDAC patient treated with pRAMPS. It has been proposed that unilateral adrenalectomy does not induce adrenal insufficiency [15] because of the postoperative compensatory function achieved by the contralateral adrenal gland [16,17]. Notably, however, it has been reported that chronic adrenal insufficiency is a possible complication of unilateral adrenalectomy [18]. Mitchell et al. also reported that 22% of patients without preoperative cortisol hypersecretion develop adrenal insufficiency after unilateral adrenalectomy [19]. This is an important consideration when deciding whether to perform aRAMPS or pRAMPS for left-sided PDAC.

Our study observations indicated that the mean clinical tumor diameters, periadrenal infiltration rates, and proportions of patients receiving neoadjuvant therapy were significantly different between the aRAMPS and pRAMPS patient groups. This difference in demographics can be explained by the tendency to try pRAMPS in patients with periadrenal infiltration. We speculate that these differences could be attributable to a positive correlation between pancreatic tumor size and the probablity of adrenal gland invasion, periadrenal infiltration, and major vessel invasion in the pancreatic body. These results further suggest that our pRAMPS patients had more advanced cancers than the aRAMPS group. Hence, for appropriate oncologic and survival outcome comparisons between aRAMPS and pRAMPS, we needed to perform subgroup comparisons including only patients without evidence of periadrenal infiltration on preoperative imaging.

The postoperative complication rate, POPF rate, mean hospitalization duration, and 90-day mortality rate were not significantly different between the aRAMPS and pRAMPS groups. However, the mean operation time was longer in the pRAMPS group, and fewer patients who underwent pRAMPS were treated with minimally invasive surgery. Adrenalectomy among the pRAMPS patients did not cause more complications or adrenal insufficiency, suggesting that pRAMPS is a feasible alternative to aRAMPS. To our knowledge, although there are several reports that the RAMPS technique is feasible compared with SDP, there have been no prior studies that have focused on pRAMPS only. Park et al. reported that the complication rates are comparable between RAMPS and SDP [5]. Trottman et al. also reported that the complication rates and grades are no different between RAMPS and SDP [20]. Randomized clinical trials to compare SDP, aRAMPS, and pRAMPS are warranted to shed further light on this, but our observations provide solid evidence that pRAMPS is a feasible surgical method.

We found that the pathologic outcomes were not different between the two RAMPS groups either before or after the exclusion of patients with periadrenal infiltration cases. The number of harvested lymph nodes, resection margin status, and tangential margin status were not significantly different. Many previous studies have reported that RAMPS has benefits in terms of the number of harvested lymph nodes and R0 resection success when compared with SDP [3,13,20,21], but there was no difference between aRAMPS and pRAMPS in this regard in our analysis. Additionally, only 5.8% of the patients in our series treated with pRAMPS actually had histopathologically confirmed adrenal invasion, and only 0.6% of the patients had histopathologically confirmed adrenal invasion in the pRAMPS group after excluding patients with periadrenal infiltration or adrenal invasion suspected after preoperative imaging analyses. This can be explained by the anterior renal fascia, which is in front of the adrenal gland and acts as a barrier against PDAC invasion. Kitagawa et al. reported that 73% of PDACs infiltrate posteriorly beyond the parenchyma but within the fusion fascia that lies between the pancreas and retroperitoneal organ and that only 3% of PDACs infiltrate the retroperitoneal tissues beyond the fusion fascia [22]. Hence, it is not necessary to perform pRAMPS to achieve a negative tangential resection margin, and a sufficient harvested lymph node number in all left-sided PDAC patients, i.e., with aRAMPS alone, will likely secure appropriate histopathologic outcomes in the absence of adrenal gland invasion or periadrenal infiltration.

We found that the OS and DFS outcomes were comparable between the aRAMPS and pRAMPS groups. Abe et al. reported that RAMPS is associated with a tendency toward improved median survival times when compared with SDP, but this was not a statistically significant difference [3]. Kim et al. also presented data demonstrating no significant difference in the 3-year DFS or OS between RAMPS and SDP [4]. Previous meta-analyses have reported, however, that the 1-year OS rate is higher in association with RAMPS compared with SDP [14] and that the recurrence rate is significantly lower in RAMPS patients [21]. The benefits, in terms of survival outcomes following RAMPS, remain unclear. However, in several studies, RAMPS has been associated with better R0 resection rates and lymph node retrieval yields compared with SDP. In contrast, it is understandable that there would be no survival differences between aRAMPS and pRAMPS if they have comparable histopathologic outcomes, and a randomized controlled trial would ultimately clarify this.

Our study had several limitations. The retrospective data collection, single-center design, and non-randomized nature of the analysis made the results prone to selection bias and confounders. As mentioned above, the selection criteria for SDP, aRAMPS, and pRAMPS were not clearly distinguished. Eight surgeons participated in the current study, and although all surgeons selected the proper operation method that can achieve R0 resection, there was a difference in the detailed selection criteria. In addition, the most important consideration in choosing a surgical method is to clearly understand the patient’s status. Therefore, the interpretation of the current study should be carefully done. Nevertheless, this study is meaningful because the purpose of this study is not to assume that pRAMPS would be superior to aRAMPS but to determine whether there is a difference between the two procedures by comparison. To the best of our knowledge, a previous large-scale comparison between aRAMPS and pRAMPS has not been conducted. We presented statistical evidence, through robust pathological results and survival analyses, that pRAMPS is not required for left-sided PDAC in the absence of adrenal gland invasion or periadrenal infiltration. In addition, based on the current study, multi-center research should be carried out in various countries, and randomized clinical trials are necessary.

## 5. Conclusions

Posterior RAMPS is a feasible and safe alternative to aRAMPS for left-sided PDAC but has no advantages over aRAMPS in terms of histopathologic outcomes, including a negative tangential resection rate and the number of harvested lymph nodes, if there is no adrenal gland invasion or periadrenal infiltration. Hence, if there is no suspicion of adrenal gland invasion in a patient with left-sided PDAC, aRAMPS alone will be an effective surgical option.

## Figures and Tables

**Table 1:** Demographic and clinical characteristics of patients who underwent radical antegrade modular pancreatosplenectomy.

Variable		aRAMPS (*n* = 112)	pRAMPS (*n* = 224)	*p*-Value
Age, years (±SD)	Mean	61.8 (±10.3)	62.7 (±9.5)	0.426
Sex, *n* (%)	Female	44 (39.3)	112 (50.0)	0.063
	Male	68 (60.7)	112 (50.0)	
BMI, kg/m^2^ (±SD)	Mean	23.9 (±2.8)	23.6 (±2.9)	0.462
ASA score, *n* (%)	I	8 (7.1)	17 (7.6)	0.281
	II	95 (84.8)	198 (88.4)	
	III	9 (8.0)	9 (4.0)	
CA 19-9, *n* (%)	Normal	47 (42.0)	93 (41.5)	0.727
	Increased	60 (53.6)	129 (57.6)	
	NA	5 (4.5)	2 (0.1)	
CEA, *n* (%)	Normal	86 (76.8)	180 (80.4)	0.687
	Increased	20 (17.9)	37 (16.5)	
	NA	6 (5.4)	5 (2.2)	
mGPS, *n* (%)	0	95 (84.8)	174 (77.7)	0.188
	1-2	5 (4.5)	18 (8.0)	
	NA	12 (10.7)	32 (14.3)	
Clinical tumor size, cm (±SD)	Mean	2.8 (±1.4)	3.3 (±1.7)	0.021
Tumor location	Neck	2 (1.8)	1 (0.4)	0.317
	Body	54 (48.2)	121 (54.0)	
	Tail	56 (50.0)	102 (45.5)	
Periadrenal infiltration	Yes	6 (5.4)	67 (29.9)	<0.001
	No	106 (94.6)	157 (70.1)	
Neoadjuvant, *n* (%)	Yes	8 (7.1)	36 (16.1)	0.022
	No	104 (92.9)	188 (83.9)	

SD, standard deviation; BMI, body mass index; NA, not available; mGPS, modified Glasgow prognostic score.

**Table 2:** Perioperative outcomes of radical antegrade modular pancreatosplenectomy.

Variables		aRAMPS (*n* = 112)	pRAMPS (*n* = 224)	*p*-Value
Operation time, minutes (±SD)	Mean	204 (±61)	228 (±63)	0.001
Operation type	Minimal invasive	80 (71.4)	122 (54.5)	0.003
	Open	32 (28.6)	102 (45.5)	
Concurrent vessel resection, *n* (%)	Yes	6 (5.4)	30 (13.4)	0.025
	No	106 (94.6)	194 (86.6)	
Concurrent resection of other organs, *n* (%)	Yes	19 (17.0)	49 (21.9)	0.291
	No	93 (83.0)	175 (78.1)	
Hospital stay after operation, days(±SD)	Mean	10.9 (±6.5)	11.5 (±7.0)	0.422
Complications grade ^+^, *n* (%)	No	86 (76.8)	164 (73.5)	0.97
	Grade I-II	12 (10.7)	39 (17.5)	
	Grade III-IV	14 (12.5)	20 (9.0)	
POPF ^++^, *n* (%) ^+^	No or	97 (86.6)	195 (87.1)	0.909
	Biochemical leakage			
	Grade B or C	15 (13.4)	29 (12.9)	
90-day mortality, *n* (%)	Yes	1 (0.9)	1 (0.4)	1
	No	111 (99.1)	223 (99.6)	
Adjuvant	No	26 (23.2)	64 (28.6)	0.522
	CTx	68 (60.7)	123 (54.9)	
	CCRTx	18 (16.1)	35 (15.6)	
	RT	0 (0.0)	2 (0.9)	
Interval between surgery and adjuvant treatment, days (±SD)	Mean	42.5 (±21.5)	44.1 (±28.6)	0.673
First line adjuvant chemotherapy regimen	Fluoropyrimidine	32 (37.6)	64 (40.5)	0.235
	Gemcitabine based	46 (53.5)	60 (38.0)	
	FOLFIRONOX	1 (1.2)	11 (7.0)	
	Others	7 (8.2)	23 (14.6)	

^+^ The complication grade was classified according to the Clavien–Dindo classification system^7^. ^++^ The POPF was graded according to the definition updated in 2016 by the International Study Group for Pancreatic Fistula. SD, standard deviation; POPF, postoperative pancreatic fistula; CTx, chemotherapy; CCRTx, concurrent chemoradiation therapy; RT, radiation therapy; FOLFIRINOX, fluorouracil, leucovorin, irinotecan, and oxaliplatin.

**Table 3:** Pathologic outcomes of radical antegrade modular pancreatosplenectomy.

Variables		aRAMPS (*n* = 112)	pRAMPS (*n* = 224)	*p*-Value
Pathologic tumor size, cm (±SD)	Mean	3.4 (±1.6)	3.8 (±1.8)	0.128
T stage (AJCC 8th), *n* (%)	T1	20 (17.9)	27 (12.1)	0.173
	T2	58 (51.8)	118 (52.7)	
	T3	33 (29.5)	77 (34.4)	
	T4	1 (0.9)	2 (0.9)	
N stage (AJCC 8th), *n* (%)	N0	50 (44.6)	83 (37.2)	0.263
	N1	48 (42.9)	109 (48.9)	
	N2	14 (12.5)	31 (13.9)	
Staging (AJCC 8th) ^+^, *n* (%)	IA	14 (12.5)	15 (6.7)	0.078
	IB	21 (18.8)	44 (19.6)	
	IIA	12 (10.7)	19 (8.5)	
	IIB	48 (42.9)	100 (44.6)	
	III	14 (12.5)	29 (12.9)	
	IV	3 (2.7)	16 (7.1)	
	NA	0 (0.0)	1 (0.4)	
Differentiation	WD	11 (9.8)	15 (6.7)	0.817
	MD	80 (71.4)	170 (75.9)	
	PD	16 (14.3)	27 (12.1)	
	Others	3 (2.7)	7 (3.1)	
	NA	2 (1.8)	5 (2.2)	
Peripancreatic infiltration, *n* (%)	Yes	106 (94.6)	210 (93.8)	0.744
	No	6 (5.4)	14 (6.3)	
Lymphovascular invasion, *n* (%)	Yes	58 (51.8)	127 (56.7)	0.394
	No	54 (48.2)	97 (43.3)	
Perineural invasion, *n* (%)	Yes	88 (78.6)	186 (83.0)	0.32
	No	24 (21.4)	38 (17.0)	
Number of harvested lymph nodes, *n* (±SD)	Mean	14.8 (±8.9)	16.9 (±9.7)	0.059
Number of positive lymph nodes, *n* (±SD)	Mean	1.4 (±1.9)	1.7 (±2.1)	0.272
Positive lymph node ratio, % (±SD)	Mean	11.2 (±15.0)	10.7 (±13.3)	0.771
Resection margin ^++^, *n* (%)	R0	86 (76.8)	173 (77.2)	0.927
	R1	26 (23.2)	51 (22.8)	
Location of margin involved, *n* (%)	Pancreas margin	1 (3.8)	1 (2.0)	0.115
	Tangential margin	23 (88.5)	50 (98.0)	
	Both	2 (7.7	0 (0.0)	
Pathologic adrenal gland invasion, *n* (%)	Yes		13 (5.8)	NA
	No		211 (94.2)	

^+^ The TNM stage was graded according to the American Joint Committee on Cancer Staging Manual, 8th edition. ^++^ If the closest safe resection margin was less than 1 mm, it was categorized as R1. WD, well differentiated; MD, moderately differentiated; PD, poorly differentiated; NA, not available.

**Table 4:** Demographic and clinical characteristics of patients who underwent radical antegrade modular pancreatosplenectomy without periadrenal infiltration.

Variables		aRAMPS (*n* = 106)	pRAMPS (*n* = 157)	*p*-Value
Age, years (±SD)	Mean	62.1 (±10.3)	62.1 (±9.2)	0.999
Sex, *n* (%)	Female	44 (41.5)	82 (52.2)	0.088
	Male	62 (58.5)	75 (47.8)	
BMI, kg/m^2^ (±SD)	Mean	23.9 (±2.9)	23.8 (±2.9)	0.888
ASA score, *n* (%)	I	8 (7.5)	13 (8.3)	0.276
	II	90 (84.9)	139 (88.5)	
	III	8 (7.5)	5 (3.2)	
CA 19-9, *n* (%)	Normal	44 (41.5)	67 (42.7)	0.888
	Increased	57 (53.8)	90 (57.3)	
	NA	5 (4.7)	0 (0.0)	
CEA, *n* (%)	Normal	83 (78.3)	128 (81.5)	0.89
	Increased	17 (16.0)	25 (15.9)	
	NA	6 (5.7)	4 (2.5)	
mGPS, *n* (%)	0	90 (84.9)	126 (80.3)	0.567
	1-2	4 (3.8)	9 (5.7)	
	NA	12 (11.3)	22 (14.0)	
Clinical tumor size, cm (±SD)	Mean	2.8 (±1.4)	2.9 (±1.3)	0.778
Tumor location	Neck	2 (1.9)	1 (0.6)	0.102
	Body	53 (50.0)	99 (63.1)	
	Tail	51 (48.1)	57 (36.3)	
Neoadjuvant, *n* (%)	Yes	8 (7.5)	23 (14.6)	0.08
	No	98 (92.5)	134 (85.4)	

SD, standard deviation; BMI, body mass index; NA, not available; mGPS, modified Glasgow prognostic score.

**Table 5:** Perioperative outcomes of radical antegrade modular pancreatosplenectomies among patients without periadrenal infiltration.

Variables		aRAMPS (*n* = 106)	pRAMPS (*n* = 157)	*p*-Value
Operation time, minutes (±SD)	Mean	202 (±61)	226 (±62)	0.002
Operation type	Minimally invasive	75 (70.8)	93 (59.2)	0.056
	Open	31 (29.2)	64 (40.8)	
Concurrent vessel resection, *n* (%)	Yes	6 (5.7)	23 (14.6)	0.022
	No	100 (94.3)	134 (85.4)	
Concurrent resection of other organs, *n* (%)	Yes	16 (15.1)	25 (15.9)	0.856
	No	90 (84.9)	132 (84.1)	
Hospital stay after operation, days (±SD)	Mean	10.8 (±6.6)	11.0 (±6.0)	0.786
Complications grade ^+^, *n* (%)	No	81 (76.4)	114 (72.6)	0.782
	Grade I-II	12 (11.3)	26 (16.6)	
	Grade III-IV	13 (12.3)	17 (10.8)	
POPF ^++^, *n* (%) ^+^	No or	92 (86.8)	135 (86.0)	0.852
	Biochemical leakage			
	Grade B or C	14 (13.2)	22 (14.0)	
90-day mortality, *n* (%)	Yes	1 (0.9)	0 (0.0)	0.403
	No	105 (99.1)	157 (100.0)	
Adjuvant	No	25 (23.6)	41 (26.1)	0.647
	CTx	64 (60.4)	91 (58.0)	
	CCRTx	17 (16.0)	23 (14.6)	
	RT	0 (0.0)	2 (1.3)	
Interval between surgery and adjuvant treatment, days (±SD)	Mean	42.9 (±21.7)	43.7 (±32.8)	0.867
First line adjuvant chemotherapy regimen	Fluoropyrimidine	30 (37.0)	46(40.4)	0.125
	Gemcitabine based	44 (54.3)	41 (36.0)	
	FOLFIRONOX	1 (1.2)	7 (6.1)	
	Others	6 (7.4)	20 (17.5)	

^+^ The complication grade was classified according to the Clavien–Dindo classification system^7^. ^++^ The POPF was graded according to the definition updated in 2016 by the International Study Group for Pancreatic Fistula. SD, standard deviation; POPF, postoperative pancreatic fistula; CTx, chemotherapy; CCRTx, concurrent chemoradiation therapy; RT, radiation therapy; FOLFIRINOX, fluorouracil, leucovorin, irinotecan, and oxaliplatin.

**Table 6:** Pathologic outcomes of radical antegrade modular pancreatosplenectomy among patients without periadrenal infiltration.

Variables		aRAMPS (*n* = 106)	pRAMPS (*n* = 157)	*p*-Value
Pathologic tumor size, cm (±SD)	Mean	3.4 (±1.7)	3.4 (±1.5)	0.909
T stage (AJCC 8th), *n* (%)	T1	20 (18.9)	22 (14.0)	0.694
	T2	55 (51.9)	91 (58.0)	
	T3	30 (28.3)	43 (27.4)	
	T4	1 (0.9)	1 (0.6)	
N stage (AJCC 8th), *n* (%)	N0	46 (43.4)	61 (39.1)	0.725
	N1	47 (44.3)	78 (50.0)	
	N2	13 (12.3)	17 (10.9)	
Staging (AJCC 8th) ^+^, *n* (%)	IA	14 (13.2)	12 (7.6)	0.314
	IB	19 (17.9)	35 (22.3)	
	IIA	11 (10.4)	11 (7.0)	
	IIB	47 (44.3)	72 (45.9)	
	III	13 (12.3)	18 (11.5)	
	IV	2 (1.9)	8 (5.1)	
	NA	0 (0.0)	1 (0.6)	
Differentiation	WD	11 (10.4)	13 (8.3)	0.558
	MD	75 (70.8)	123 (78.3)	
	PD	15 (14.2)	16 (10.2)	
	Others	3 (2.8)	2 (1.3)	
	NA	2 (1.9)	3 (1.9)	
Peripancreatic infiltration, *n* (%)	Yes	100 (94.3)	147 (93.6)	0.813
	No	6 (5.7)	10 (6.4)	
Lymphovascular invasion, *n* (%)	Yes	54 (50.9)	87 (55.4)	0.476
	No	52 (49.1)	70 (44.6)	
Perineural invasion, n (%)	Yes	82 (77.4)	127 (80.9)	0.487
	No	24 (22.6)	30 (19.1)	
Number of harvested lymph nodes, *n* (±SD)	Mean	15.1 (±9.0)	17.0 (±9.9)	0.118
Number of positive lymph nodes, *n* (±SD)	Mean	1.4 (±1.8)	1.5 (±2.0)	0.675
Positive lymph node ratio, % (±SD)	Mean	11.0 (±13.9)	9.6 (±11.5)	0.414
Resection margin ^++^, *n* (%)	R0	82 (77.4)	127 (80.9)	0.487
	R1	24 (22.6)	30 (19.1)	
Location of margin involved, *n* (%)	Pancreas margin	1 (4.2)	1 (3.3)	0.519
	Tangential margin	22 (91.7)	29 (96.7)	
	Both	1 (4.2)	0 (0.0)	
Pathologic adrenal gland invasion, *n* (%)	Yes		1 (0.6)	NA
	No		156 (99.4)	

^+^ The TNM stage was graded according to the American Joint Committee on Cancer Staging Manual, 8th edition. ^++^ If the closest safe resection margin was less than 1 mm, it was categorized as R1. WD, well differentiated; MD, moderately differentiated; PD, poorly differentiated; NA, not available.

**Table 7:** Oncologic outcomes of radical antegrade modular pancreatosplenectomy among patients without periadrenal infiltration.

Variables		aRAMPS (*n* = 106)	pRAMPS (*n* = 157)	*p*-Value
Time to recurrence, months	Median	12	13.3	0.534
Site of recurrence	Local	10 (13.0)	24 (20.9)	0.463
	Systemic	35 (45.5)	50 (43.5)	
	Local + systemic	10 (13.0)	16 (13.9)	
	Peritoneal carcinomatosis	22 (28.6)	25 (21.7)	
Median overall survival, months	Median	30.3	32.8	0.318
4-year survival rate, (%)		34.7	35.1	0.947

## Data Availability

The data presented in this study are available on request from the corresponding author. The data are not publicly available due to private information of patients.
